# News and Events of the Week

**Published:** 1909-10-16

**Authors:** 


### News and Events of the Week.

Dr. R. A. Forsyth, after having been medical officer for the Birstal district of the Dewsbury Board of Guardians for twenty-seven years, has resigned his post.

At the Cancer Hospital, Fulham Road, Miss Constance Davys and friends kindly entertained the in-patients on last week, when *Niobe* was performed. The piece was capitally acted and gave great enjoyment.

At the fortnightly meeting of the Barnet Board of Guardians the resignation was accepted of Dr. Thomas Thyne, medical officer of the workhouse and public vaccinator. Dr. Thyne has been medical officer for twenty-four years.

In London 2,220 births and 1,127 deaths were registered during the week ending September 25. The annual death-rate from all causes, which in the previous week was at the rate of 12.4 per 1,000, fell to 12.2. The total births were three and the deaths 34 fewer than in the previous week.

Amongst the latest contributions received for King Edward's Hospital Fund for London are the following :—Annual subscriptions : Alderman Sir W. Vaughan Morgan, Bart., £50; Rev. A. W. Davies, £25; Messrs. Tattersall, £25. Donations : Mr. C. W. Drabble, £105; Home and Colonial Stores, Ltd., £26 5s.

A meeting in support of the League of Mercy will be held at the Mansion House on Thursday, the 21st inst., at 4 o'clock. The Lord Mayor will preside. Lady Dimsdale, recently appointed lady president of the City branch, will take steps to make the League of Mercy better supported among the banking and mercantile community of the City of London.

In Greater London the births during the week ending September 25 were 3,477 and the deaths 1,628. These numbers are 182 and 271 below the respective averages in the corresponding weeks of the last five years. The death-rate was 11.4 per 1,000, as against 11.6 in the previous week. The total births were 54 and the deaths 28 fewer than according to the previous return.

At a meeting of Dundee District Committee of Forfar County Council the Medical Officer of Health reported that an outbreak of diphtheria could be traced to the polluted and dangerous state of a burn in the Downfield district. It was explained that the Dundee authorities were responsible for the state of affairs, and that the nuisance was being aggravated by the fact that Downfield sewage meadow was not being used.

The twenty-second French Congress of Surgery was opened on October 4th at the Faculty of Medicine in Paris. Dr. Richelot, in his presidential address on the progress of surgery during the last quarter of a century, quoted the words of Velpeau, "Sans l'érysipèle et l'infection purulente les chirurgiens seraient des dieux," and added : "These accidents have disappeared, but we are still men. Yes, surgery is constantly making progress, but what obstacles still lie in its path ! And I do not refer to the charlatans. There is an immense quantity of undigested literature which renders slight service to our patients. I, for my part, love *la chirurgie simple*."

Mr. Mansell-Moullin, senior surgeon at the London Hospital, who retires to the consulting staff this month, has completed twenty years on the senior staff, after being assistant surgeon for seven years, previous to which he was surgical registrar.

The proceeds of the Birmingham Musical Festival have been handed to the General Hospital Funds. A hygienic exhibition was opened last week at Carlton Buildings, Paradise Street, Birmingham, by Sir William Bennett. Sir Thomas Barclay occupied the chair, supported by many well-known people interested in hygiene.

The annual meeting and festival of the Birmingham Ward of the Guild of St. Luke, will be held on Wednesday, October 27, at 8 p.m., at Holy Trinity Church, Coventry, when the festival sermon will be preached by the Lord Bishop of Worcester. All medical practitioners in the district are invited to attend.

A course of lectures on "First Aid to the Injured, and Nursing" was inaugurated last week at the Regent Street Polytechnic, the course being intended for ladies who desire to qualify for the voluntary aid section in connection with the British Red Cross Society as sanctioned by the War Office. These sections will form the nursing staff of the various Territorial regiments, and the ladies composing them are required to pass an examination on the subjects of first aid and nursing. The course is being delivered by Dr. James Cantlie, who, at the first lecture, had an audience of upwards of one hundred ladies.

An inquest was held at Shoreham on October 2 on the body of a retired master mariner. The medical evidence was to the effect that the deceased had a large, irritable, and inflamed ulcer between the middle and fourth toe of the left foot, the result of applying a corn solvent to an old corn. The ulcer spread, and it was found necessary to amputate the toe. The wound showed little tendency to heal, and the tendons of the foot became gangrenous, and the man died from acute septicæmia. The corn solvent used was one of numerous preparations containing salicylic acid, which are strongly corrosive. The jury found that death was due to blood poisoning set up by the use of a corrosive liquid, as a corn solvent, and the Coroner said the danger of using these solvents could not be too widely known, and those who had the selling of them should caution people regarding them.

In connection with the Chadwick Training Course in School Hygiene, Professor Henry Kenwood delivered at University College a public introductory lecture on "What Hygiene Demands of School Teachers." Sir James Crichton-Browne presided. Dr. Kenwood said it was only quite recently that the Board of Education had made school hygiene a compulsory subject in training colleges. There was a tendency among those school teachers who had devoted time and study to the subject to undue scientific elabortion of it. It was to be regretted that school baths were not more frequently provided. In some towns in Germany a large proportion of the schools had their baths. Hygienists were concentrating their hopes upon the schools, for it was recognised that the next great development of hygiene throughout the community must depend upon school influences.

The Duchess of Albany will visit St. Peter's Hall, Brockley, on Saturday, the 23rd inst., in connection with a sale of work and meeting organised by the Deptford District of the League of Mercy, of which her Royal Highness is president.

---

A very good photograph of Hogarth's picture of the Pool of Bethesda, which decorates the staircase leading up to the Great Hall, at St. Bartholomew's Hospital, has been taken by Messrs. Haufstaengel of Pall Mall. The photograph was only obtained under great difficulty owing to the situation of the picture on the wall in a very bad light, and it required three hours' exposure on a bright sunny morning in midsummer.

---

At the otological meeting at the International Medical Congress, which has just been held in Budapest, the Leswal prize was divided between Dr. Gray of Glasgow, and Professor Heinrich Neumann, of Vienna. Professor Neumann's work has consisted in the application to aural surgery of the infiltration method of anæsthesia, while Dr. Gray's studies on the anatomy of the internal ear have led to his being looked upon as an authority on the subject.

---

On being asked last week the number of soldiers discharged from the Army during the ten years ending 1908 who suffered from tuberculosis, Mr. Haldane gave the following figures : 1899, 224; 1900, 328; 1901, 350; 1902, 334; 1903, 301; 1904, 440; 1905, 333; 1906, 400; 1907, 304; 1908, 272. While in hospital, he added, patients suffering from tubercle of the lung are kept apart from other patients and treated generally as cases of infectious disease. On discharge a notification is sent to the medical officer of health in the town or district where the man proposes to reside.

---

Dr. H. Cooper Pattin presided at the fifty-third annual dinner of the Society of Medical Officers of Health, held on October 7 at the Criterion Restaurant. Sir Robert Morant, Secretary of the Board of Education, in proposing the toast of the evening, said they had to a considerable extent been successful in linking up the machinery of health and education, and the public was coming to recognise the importance of prevention rather than palliation. A great part of their business was to remove conditions which made it possible for people to become unhealthy. By their new opportunities of inspection medical officers now had great powers, which he hoped would result in great good to the race.

---

The following is the programme arranged by the Society of Tropical Medicine and Hygiene for the ensuing session :—October 15 : A discussion on Sleeping Sickness will be opened by Dr. Arthur G. Bagshawe, who will read a paper on "Recent Advances in our Knowledge of that Disease." November 19 : A paper (with specimens) will be read by D. J. Numa Rat, St. Kitts, W.I., on "The So-called Guinea-worm of the Island of Nevis." December 17 : A discussion on Tick Fever will be opened by Sir W. B. Leishman. January 21 : A discussion on Ankylostomiasis will be opened by Sir Thomas Oliver, Newcastle-on-Tyne; and a paper on "Ankylostomiasis in Natal" will be read by Dr. John J. Elliott. February 18 : A discussion on Medical Entomology will be opened by Mr. E. E. Austen, who will read a paper entitled "Some Dipterous Insects which Cause Myiasis in Man." March 18 : A discussion on Helminthology will be opened by Dr. J. W. W. Stephens, and papers from Dr. Houghton, of Wuhu, and others will be read.

